# A Referral Center Experience with Cerebral Protection Devices: Challenging Cardiac Thrombus in the EP Lab

**DOI:** 10.3390/jcm12041549

**Published:** 2023-02-16

**Authors:** Jan Berg, Alberto Preda, Nicolai Fierro, Alessandra Marzi, Andrea Radinovic, Paolo Della Bella, Patrizio Mazzone

**Affiliations:** 1 Division of Arrhythmology, San Raffaele Hospital, 20132 Milan, Italy; 2 Division of Cardiology, Cantonal Hospital of Aarau, 5001 Aarau, Switzerland

**Keywords:** left atrial appendage closure, catheter ablation, stroke, cerebral protection

## Abstract

BACKGROUND: Cerebral protection devices (CPD) are designed to prevent cardioembolic stroke and most evidence that exists relates to TAVR procedures. There are missing data on the benefits of CPD in patients that are considered high risk for stroke undergoing cardiac procedures like left atrial appendage (LAA) closure or catheter ablation of ventricular tachycardia (VT) when cardiac thrombus is present. PURPOSE: This work aimed to examine the feasibility and safety of the routine use of CPD in patients with cardiac thrombus undergoing interventions in the electrophysiology (EP) lab of a large referral center. METHODS: The CPD was placed under fluoroscopic guidance in all procedures in the beginning of the intervention. Two different CPDs were used according to the physician’s discretion: (1) a capture device consisting of two filters for the brachiocephalic and left common carotid arteries placed over a 6F sheath from a radial artery; or (2) a deflection device covering all three supra-aortic vessels placed over an 8F femoral sheath. Retrospective periprocedural and safety data were obtained from procedural reports and discharge letters. Long-term safety data were obtained by clinical follow-up in our institution and telephone consultations. RESULTS: We identified 30 consecutive patients in our EP lab who underwent interventions (21 LAA closure, 9 VT ablation) with placement of a CPD due to cardiac thrombus. Mean age was 70 ± 10 years and 73% were male, while mean LVEF was 40 ± 14%. The location of the cardiac thrombus was the LAA in all 21 patients (100%) undergoing LAA-closure, whereas, in the 9 patients undergoing VT ablation, thrombus was present in the LAA in 5 cases (56%), left ventricle (n = 3, 33%) and aortic arch (n = 1, 11%). The capture device was used in 19 out of 30 (63%) and the deflection device in 11 out of 30 cases (37%). There were no periprocedural strokes or transitory ischemic attacks (TIA). CPD-related complications comprised the vascular access and were as follows: two cases of pseudoaneurysm of the femoral artery not requiring surgery (7%), 1 hematoma at the arterial puncture site (3%) and 1 venous thrombosis (3%) resolved by warfarin. At long-term follow-up, 1 TIA and 2 non-cardiovascular deaths occurred, with a mean follow-up time of 660 days. CONCLUSIONS: Placement of a cerebral protection device prior to LAA closure or VT ablation in patients with cardiac thrombus proved feasible, but possible vascular complications needed to be taken into account. A benefit in periprocedural stroke prevention for these interventions seemed plausible but has yet to be proven in larger and randomized trials.

## 1. Introduction

Stroke following electrophysiologic (EP) interventions of the heart is a serious complication. Periprocedural stroke risk is estimated between 0.1–0.9% in patients undergoing catheter ablation of atrial fibrillation [1,2,3,4], between 0.8–1.8% in patients undergoing ablation of ventricular tachycardia (VT) [4,5,6] and 0.7–1.1% in patients undergoing endocardial appendage closure [7,8]. Therefore, it is crucial to reduce stroke risk in patients that are considered high risk of periprocedural stroke. However, strategies to avoid periprocedural stroke are not well established in the general EP lab. Recently, cerebral protection devices (CPD) have become commercially available; the two most frequently used systems are the Sentinel © (Boston Scientific, Marlborough, MA, USA) and the Triguard © system (Keystone Heart, Caesarea, Israel). These two CPD differ in deployment and arrangement, and both systems have been widely studied in transcatheter aortic valve replacement (TAVR) procedures [9,10,11,12,13,14,15].

However, there are insufficient data about the benefits of CPD in EP procedures. To our knowledge, there have been two small studies with CPD in patients undergoing ablation of ventricular tachycardia. The first study reported feasibility and safety in a series of 11 patients with ischemic heart disease undergoing VT ablation [16] using the Sentinel© CPD device. The authors reported detection of debris in the device after the procedure in all patients, highlighting the plausibility of using CPDs in patients undergoing procedures with elevated stroke risk. In a second study, our group reported on the feasibility and safety of using the Sentinel and Triguard CPD in 7 patients undergoing ablation of ventricular tachycardia [17]. Recently, the feasibility of left atrial appendage (LAA) closure in presence of a cardiac thrombus was reported for the Sentinel [18,19] and the Triguard device [20].

The aim of this study was to provide data on the feasibility of using the two most common CPD in patients with cardiac thrombus undergoing cardiac interventions in the EP lab of a large tertiary center—and to report periprocedural and long-term outcomes in this selected group.

## 2. Materials and Methods

We conducted a single-center retrospective and observational study, including all patients undergoing procedures with deployment of a CPD, in the EP labs of the Arrhythmology Department at San Raffaele Hospital, Milan, Italy between June 2016 and April 2022. A total of 32 consecutive patients were identified; 2 had to be excluded because of insufficient data. The study was conducted according to institutional guidelines and legal requirements and complied with the Declaration of Helsinki.

### 2.1. Clinical and Echocardiographic Data

Baseline clinical data were retrospectively collected by clinical chart review. All patients underwent baseline transthoracic echocardiography (TTE). Transoesophageal echocardiography (TOE) was performed in all patients with planned endocardial LAA closure. During TOE, thrombotic material was classified as either sludge or manifest thrombus. TTE and TOE were conducted with a Vivid E95 (GE healthcare, USA) and a M5Sc probe for TTE and 6VT-D probe for TOE.

### 2.2. Procedures

All procedures were conducted in general anesthesia. The CPD was placed under fluoroscopic guidance in all procedures in the beginning of the procedure, before a transseptal puncture. There was one experienced operator for LAA closure and one experienced operator for VT ablation, respectively, who were responsible for the main procedure and placement of the protection device. Both operators were assisted by at least one experienced electrophysiologist or fellow during the procedure.

#### 2.2.1. Sentinel Device

**Figure 1:**
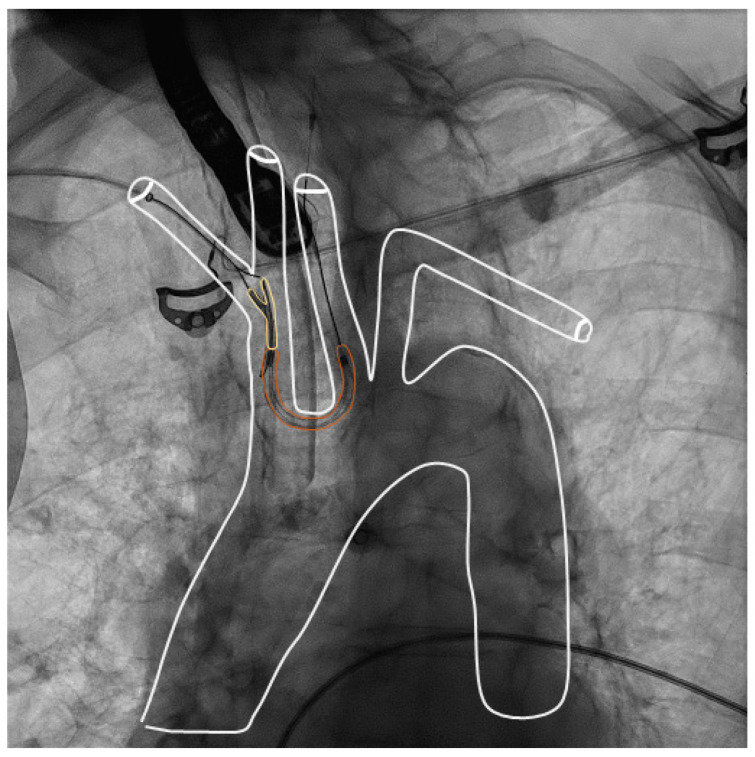
The Sentinel device in standard position, covering the brachiocephalic and left common carotid artery. Anteroposterior view. Aortic arch and supraaortic vessels are depicted in white, the device is depicted in yellow for better visibility.

The Sentinel CPD is a capture device consisting of two polyurethane filters with 140-µm-diameter pores designed to capture thrombotic material. It is inserted from a radial or brachial artery over a 6F delivery catheter with a deflectable distal tip. The proximal filter is placed in the brachiocephalic trunk, the distal filter is placed in the left common carotid artery (Figure 1; online Supplemental Video S1). Before device placement, the aortic branches are visualized by contrast angiography over a pigtail catheter which is placed in the ascending aorta.

#### 2.2.2. Triguard 3 Device

**Figure 2:**
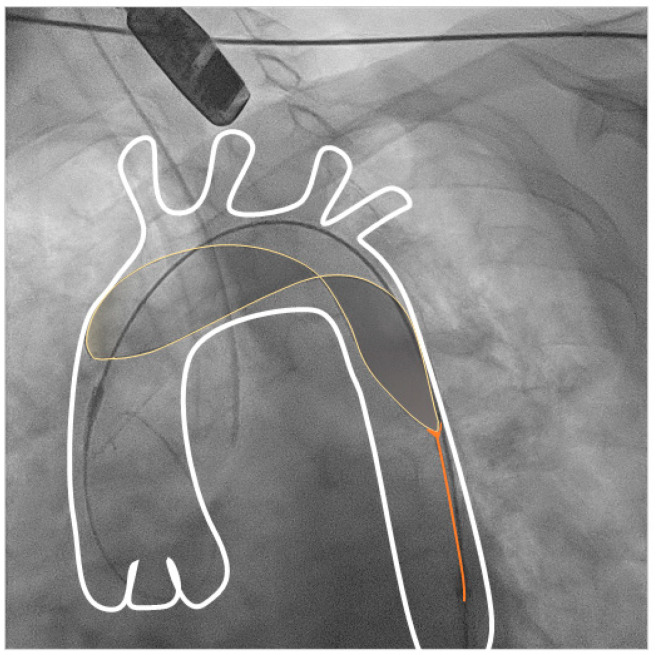
Triguard device in standard position covering the supra-aortic vessels. Anteroposterior view. Aortic arch and supraaortic vessels are depicted in white, the device is depicted in yellow for better visibility.

The Triguard 3 CPD is a deflection device which covers all three supra-aortic vessels (brachiocephalic, left common carotid and left subclavian arteries, Figure 2; online Supplemental Video S2) with a polymeric mesh, with a pore size of 115 × 145 µm. It is placed over an 8F delivery catheter from a femoral artery and designed to self-position in the aortic arch.

#### 2.2.3. LAA Closure Procedure

LAA closure was conducted in general anesthesia using no-touch implantation technique in order to avoid dislodgement of thrombotic material, as previously described by our group [18]. A single puncture of the fossa ovalis was performed from the infero-posterior site for optimal alignment with the LAA. After transseptal puncture, intravenous heparin was administered with a goal of 300 s of activated clotting time (ACT). The evaluation of the LAA anatomy to select the correct device size was performed with TOE in all cases and, only in the case of suboptimal imaging, a small dose of contrast agent was manually injected with a pigtail catheter near the LAA ostium without advancing it deeply into the LAA. The device was unsheathed and later completely deployed under TOE and fluoroscopic monitoring. Device stability was assessed with the tug test, and the result was confirmed by angiography. Small thrombotic debris were eventually captured in the CPD at the end of the procedure. Finally, the correct positioning and the absence of significant peri-device leaks were confirmed by angiography and 2D/3D TOE. After the procedure, hemostasis was reached through removal of the sheath and subsequent manual compression of the venous and arterial access sites, when the ACT was less than 180 s.

#### 2.2.4. VT Ablation Procedure

VT ablations were performed under general anesthesia using the standard approach in our institution, as published previously [21]. Programmed electrical stimulation was utilized to induce the VT. For the antegrade approach, venous access was obtained via the right femoral vein and a single transseptal puncture was performed under fluoroscopic guidance. For the retrograde approach, an 8F sheath was advanced into the descending aorta via the right femoral artery. An ACT level of 300 s was aimed during the procedure. LV mapping was conducted utilizing either the CARTO 3 (Biosense Webster, Inc.) or the ENSITE X electroanatomical mapping system (Abbott, MN, USA). The geometry of the chamber was reconstructed in sinus rhythm with the ablation catheter and then refined with a high density multipolar mapping catheter. Scar zones and local late potentials were mapped. Additional epicardial mapping and ablation was performed in patients with an ECG morphology or a substrate suggestive of an epicardial origin. If a hemodynamically stable VT could be induced, the critical isthmus of the VT was mapped with the multipolar catheter and ablated. For hemodynamically unstable VT, pace mapping was performed at different sites within the low-voltage area during sinus rhythm to identify the VT exit and potential critical isthmus. Radiofrequency current was delivered with a maximum power of 50W. The procedural end point was the elimination of late potentials and the non-inducibility of any sustained VT via programmed electrical stimulation.

### 2.3. Periprocedural Events and Follow-Up

Information about periprocedural outcomes, including all procedure and device-related adverse events (<7 days), were taken from patient charts and electronic patient files. Complications were divided into two categories: (1) related to the CPD or (2) not related to the CPD.

Additional long-term follow-up was conducted by follow-up visits in our outpatient clinic or, if not available, by telephone interviews with the patient. Main focus of long-term follow-up was to assess safety of our approach and possible complications. Routine clinical follow-up visits included a clinical anamnesis, ECG and a medical examination in our outpatient clinic. Patients, after LAA closure, underwent additional TOE after 3 to 6 months in our clinic or at the referring center. Follow-up of patients after VT ablation was scheduled according to the physician’s discretion. Telephone interviews were conducted retrospectively in patients where clinical follow-up was not available and aimed for events like vascular complications or TIA/stroke.

### 2.4. Statistical Analysis

Continuous variables are expressed as mean ± standard deviation. Categorical variables are expressed as numbers and percentage. The authors decided to report descriptive data, rather than using statistical tests, in this selected and heterogenous group, with low event rates and low patient numbers. All authors had full access to all the data in the study and have taken responsibility for its integrity and that of the data analysis.

## 3. Results

### 3.1. Clinical Characteristics

Thirty consecutive patients were included in the final analysis. Baseline characteristics are summarized in Table 1. Mean age in the entire patient cohort was 70.2 ± 10.2 (range 37–87) years and 22 out of 30 patients (73%) were male. Mean CHA2DS2VASC-score was 3.2 ± 1.4 and mean HASBLED score 2.1 ± 0.8. Mean left ventricular ejection fraction was 40.2 ± 13.6. Most patients had ischemic cardiomyopathy (n = 10, 33%) as the underlying disease. Five patients (17%) had dilated cardiomyopathy, 3 patients (10%) tachycardiomyopathy, 2 primary valvular cardiomyopathy (7%), 1 rheumatic heart disease (3%) and 1 hypertrophic cardiomyopathy (3%). Additionally, 12 patients (40%) had undergone cardiac surgery before, with valvular surgery (n = 6; 20%) being the most prevalent, followed by coronary artery bypass grafting (n = 4; 13%), left ventricular aneurysmectomy (n = 1; 3%) and myectomy (n = 1; 3%). Finally, 2 patients (7%) were pacemaker carriers, and 12 patients (40%) were carriers of an implantable cardioverter-defibrillator.

In total, 21 patients (70%) underwent LAA closure, whereas 13 of those (62%) received an Amplatzer Amulet and 8 (38%) received a Watchman FLX device. Among those 21 patients who underwent LAA closure, a manifest thrombus in the LAA was found in 16 cases (76%), whereas severe sludge in LAA was found in 5 cases (24%). The most common indication for LAA closure was persistent thrombus despite anticoagulation in 13 cases (62%), a strategy previously described by our group [18] and in a meta-analysis [20]. Other indications were: inability to take oral anticoagulation because of intracranial bleeding in 5 (24%), gastrointestinal bleeding in 2 (10%) and diffuse bleeding with anemia in 1 (5%). Atrial fibrillation (AF) was present in all patients undergoing LAA closure (21 out of 21 patients). AF pattern was persistent in 13 (62%), long-standing persistent in 2 (10%) and permanent in 6 patients (29%).

Nine patients (30%) underwent VT ablation. Among those patients, a manifest thrombus in the LAA was found in 5 (56%), a left ventricular thrombus was found in 2 (22%), severe spontaneous echo contrast in the left ventricle in 1 (11%) and mobile thrombotic material in the aortic arch in 1 (11%), prior to the intervention as a reason for using a CPD. AF was present in 7 patients (78%) undergoing VT ablation (persistent n = 3, permanent n = 3, paroxysmal n = 1).

### 3.2. Procedural Data

Placement of the CPD was feasible in all patients; the Sentinel device was used in 19 out of 30 (63%) and the Triguard 3 device in 11 out of 30 cases (37%). Mean procedure time including placement of the CPD was 103 ± 25 min for LAA closure and 246 ± 29 min for VT ablation. Mean hospitalization time was 2.9 ± 2.2 days in LAA closure and 14.3 ± 12.2 days for VT ablation. The mean number of vascular access sheaths was 3.8 ± 0.7 per procedure.

### 3.3. Periprocedural Complications

Data about periprocedural complications until discharge were available for all patients.

Periprocedural complications were noted in 3 out of 21 patients undergoing LAAC (14%). Those were (1) hematoma of the right arm at the arterial puncture site with swollen extremity in 1 patient which resolved spontaneously, (2) thrombosis of the right communal femoral vein in 1 patient which was later resolved by warfarin, and (3) pseudoaneurysm of the right femoral artery which did not require surgery. All 3 complications were categorized as possibly related to the placement of the CPD.

Periprocedural complications occurred in 4 out of 9 patients (44%) undergoing VT ablation with a CPD. One patient died in-hospital after VT ablation, not related to placement of the CPD. Other complications were cardiogenic shock with insertion of an intra-aortic balloon pump in 2 patients with VT ablation, also not related to the CPD. A pseudoaneurysm of the left superficial femoral artery not requiring surgery was noted in 1 patient, which was categorized as possibly related to the CPD.

In summary, there were 4 complications related to the CPD in 30 patients (13%). All complications comprised the vascular access site and none of the complications required surgery (Table 2).

### 3.4. Follow-Up

Long-term follow-up data were available for 26 out of 30 patients (87%) and mean follow-up time was 660 days. At long-term follow-up 1 TIA and 2 non-cardiovascular deaths (1 death because of gastric cancer 575 days after LAA closure, 1 death because of pneumonia 587 days after LAA closure) were noted during long-term follow-up.

## 4. Discussion

### 4.1. Main Study Findings

The main findings of this study were the following: (1) application of the Sentinel and Triguard CPD was feasible in all patients undergoing LAA closure and VT ablation. (2) Placement of these two different devices was safe, with vascular complications that did not require surgery as the only complications in our cohort (n = 4, 13%). (3) There were no instances of periprocedural TIA or stroke despite the presence of cardiac thrombus in all patients. Finally, long-term follow-up confirmed safety of our approach in this patient cohort at high risk for stroke.

### 4.2. Comparison with Other Studies

To our knowledge, this was the largest study to report on outcomes of patients undergoing cerebral embolic protection with two different devices in a general EP lab.

Very recently, the randomized protected TAVR trial did not find significant differences in stroke incidence after TAVR in patients with a Sentinel device during TAVR [22]. There was a stroke incidence of 2.3% in the CPD group and a lower than expected 2.9% stroke incidence in the control group. However, in the subgroup analysis, the authors found a higher proportion of disabling strokes in the group without CPD. This finding was hypothesis-generating, but it raised the question of whether embolic protection could be more beneficial in patients at highest risk for stroke, rather than in the general TAVR population. Given the large number needed to treat, of 125 patients in the protected TAVR trial, to avoid one disabling stroke, one could argue that patient selection is critical to gain any benefit in stroke protection. In our study, patients were considered high risk for development of stroke because cardiac thrombus was already present.

Prior studies with single devices, mostly the Sentinel device, have been published in LAA closure and VT ablation. Bocuzzi et al. [19] published data comprising 28 patients from 8 centers with AF and LAA thrombus undergoing LAA closure and cerebral protection with the Sentinel device. They found no strokes and no complications at the access site. A systematic review by Sharma et al. identified 58 patients with LAA thrombus who underwent LAA closure. Of those, 17 received cerebral protection with different devices. No strokes were reported, but vascular complications were not assessed. Heeger et al. [16] found that cerebral protection with the Sentinel device was feasible and safe in a series of 11 patients undergoing VT ablation. Importantly, VT ablation was associated with embolization of embolic debris, found in the device filter in all patients after VT ablation. The authors reported no strokes, in line with our findings. To our knowledge, the only case where a Triguard device was used for VT ablation was published by our own group. In the initial experience by Zachariah et al. [17] with 7 patients undergoing VT ablation, Triguard was used in 1 patient and Sentinel was used in 6 patients. Placement of all devices was feasible and there were no complications.

In summary, our findings were in line with previous studies in terms of feasibility and safety for the Sentinel and Triguard devices in LAA closure and VT ablation. In contrast to previous studies, we reported on complications at the access site, which need to be balanced against the possible benefits in stroke prevention.

### 4.3. Technical Aspects

The Sentinel CPD does not protect the left vertebral and subclavian arteries. Additionally, it requires contrast angiography of the aortic branches prior to placement and correct placement of the device requires a learning curve in our experience.

The Triguard 3 device, which covers all supra-aortic vessels with a single filter, requires less experience, in our opinion. Moreover, in our experience, contrast angiography was not always mandatory, leading to the possibility of avoiding exposition with contrast agent when necessary, as in cases with renal failure or allergy against iodine contrast agent. However, the access sheath is larger (8F femoral artery sheath in Triguard 3 versus 6F radial artery sheath in Sentinel).

We reported a relatively high number of vascular complications (4 out of 30 patients, 13%) in our cohort. Of these, 1 was a hematoma, 2 were pseudoaneurysms of the femoral artery not requiring surgery and one was a venous thrombosis which was later resolved by warfarin. Of note, these patients required a high number of sheaths (mean 3.8 sheaths per procedure) for their venous and arterial accesses, respectively. These findings highlighted the importance of a standardized protocol for venous and arterial puncture and careful consideration of the necessity for each vascular access.

### 4.4. Clinical Implications

Our findings showed that placement of a cerebral protection prior to LAA closure or VT ablation in patients with cardiac thrombus was feasible. However, vascular complications could occur. The availability of two different cerebral protection devices in the EP lab offers the possibility to treat patients at high risk for stroke undergoing LAA closure and VT ablation. In our opinion, the mere possibility of safely performing an LAA closure procedure in the presence of an intracardiac thrombus is a very important aspect. The possibility of treating VT by catheter ablation in the presence of an intraventricular thrombus, or protecting against atrial embolization, in case of DC cardioversion of untolerated VT, is also very important, since it opens the possibility of safely performing potentially lifesaving procedures without adding embolic risk. The results of our single-center experience may encourage clinicians to consider cerebral protection, based on a case-to-case decision, while this approach remains unestablished in international guidelines.

### 4.5. Limitations

We did not examine the incidence of silent new cerebral embolic lesions (CEL) after intervention, since routine cMRI procedures were not performed in clinically asymptomatic patients. A previous report detected up to 32% asymptomatic CEL after Watchman implantation [23], but incidence in our selected group of high-risk patients remains speculative. Recent studies have shown frequent discovery of thrombotic debris in CPD [16], but we were unable to provide any data regarding this, as it was not systematically reported in our patient cohort.

## 5. Conclusions

Placement of a cerebral embolic protection device prior to LAA closure or VT ablation in patients with cardiac thrombus was feasible, but possible vascular complications needed to be taken into account. A benefit in periprocedural stroke prevention for these interventions seems plausible, but has yet to be proven in larger and randomized trials.

## Figures and Tables

**Table 1:** Baseline characteristics.

Variable	Total	LAA Closure	VT Ablation
No. Patients, n	30	21	9
Age, mean ± SD	70.2 ± 10.2	70.1 ± 11	70.6 ± 7.7
Female gender	8 (27%)	8 (31%)	--
			
Comorbidities			
Hypertension	16 (53%)	12 (57%)	4 (44%)
Dyslipidemia	10 (33%)	8 (38%)	2 (22%)
Diabetes Mellitus	2 (7%)	2 (10%)	--
Coronary artery disease	10 (33%)	6 (29%)	4 (44%)
			
Atrial Fibrillation, n (%)	28 (93%)	21 (100%)	7 (78%)
Paroxysmal	1 (3%)	--	1 (11%)
Persistent	16 (53%)	13 (62%)	3 (33%)
Long-standing persistent	2 (7%)	2 (10%)	--
Permanent	9 (30%)	6 (29%)	3 (33%)
Congestive Heart Failure	21 (70%)	12 (57%)	9 (100%)
PM	2 (7%)	2 (10%)	--
ICD	12 (40%)	3 (14%)	9 (100%)
Stroke or TIA history	2 (7%)	2 (10%)	--
Systemic arterial embolism	1 (3%)	1 (5%)	--
			
Bleeding predisposition	12 (40%)	10 (48%)	2 (22%)
Intracranial Hemorrhage	6 (20%)	5 (24%)	1 (11%)
GI bleeding	2 (6%)	2 (10%)	--
Diffuse bleeding with anemia	1 (3%)	1 (5%)	--
Teleangiectasia in Rendu-Osler-Weber disease	1 (3%)	1 (5%)	--
Intraretinal bleeding	1 (3%)	1 (5%)	--
Hemophilia A	1 (3%)	--	1 (11%)
			
Previous cardiac surgery	12 (40%)	7 (33%)	5 (56%)
Coronary bypass surgery	4 (13%)	3 (10%)	1 (11%)
Valvular surgery	6 (20%)	4 (13%)	2 (22%)
Myectomy	1 (3%)	--	1 (11%)
LV aneurysmectomy	1 (3%)	--	1 (11%)
			
Cardiomyopathies, n (%)	22 (73%)	13 (62%)	9 (100%)
Ischemic cardiomyopathy	10 (33%)	6 (29%)	4 (44%)
Dilated cardiomyopathy	5 (17%)	2 (10%)	3 (33%)
Primary valvular cardiomyopathy	2 (7%)	2 (10%)	--
Hypertrophic cardiomyopathy	1 (3%)	--	1 (11%)
Tachycardiomyopathy	3 (10%)	3 (14%)	--
Rheumatic heart disease	1 (3%)	--	1 (11%)
			
Risk scores			
CHA2DS2-VASc, mean ± SD	3.2 ± 1.4	3.2 ± 1.4	3.1 ± 1.4
HASBLED, mean ± SD	2.1 ± 0.8	2.1 ± 0.9	2.3 ± 0.7
			
Antithrombotic treatment	26 (84%)	17 (81%)	9 (100%)
Low dose Aspirin	4 (13%)	2 (10%)	2 (22%)
DOAC	9 (30%)	8 (38%)	1 (11%)
VKA	13 (43%)	7 (33%)	6 (67%)
			
Echo characteristics			
LVEF, mean ± SD %	40.2 ± 13.6	45.8 ± 10.1	29.6 ± 13.1
LAA thrombus or significant sludge before procedure	26 (87%)	21 (100%)	5 (56%)
LV thrombus or significant sludge before procedure	3 (10%)	--	3 (33%)
mobile thrombus in aortic arch	1 (3%)	--	1 (11%)

Abbreviations: CAD, coronary artery disease; DOAC, direct oral anticoagulant; GI, gastro intestinal; ICD, internal cardioverter-defibrillator defibrillator; LAA, left atrial appendage;; LV, left ventricle; LVEF, Left Ventricular Ejection Fraction; PM, pacemaker; SD, standard deviation; TIA: transient ischemic attack; VKA, vitamin K antagonists. Congestive Heart Failure = EF < 50%.

**Table 2:** CPD-related complications.

LAA Closure with Sentinel	LAA Closure with Triguard	VT Ablation with Sentinel	VT Ablation with Triguard
14	7	5	4
Hematoma = 1 Venous thrombosis = 1	Arterial pseudoaneurysm = 1	--	Arterial pseudoaneurysm = 1

## Data Availability

The data in this study are available on request from the corresponding author.

## Supplementary Materials

The following supporting information can be downloaded at: https://www.mdpi.com/article/10.3390/jcm12041549/s1.
